# On the Existence of Pilin-Based Microbial Nanowires

**DOI:** 10.3389/fmicb.2022.872610

**Published:** 2022-06-06

**Authors:** Derek R. Lovley

**Affiliations:** Department of Microbiology and Institute for Applied Life Sciences, University of Massachusetts, Amherst, MA, United States

**Keywords:** *Geobacter*, e-pili, extracellular electron transfer, electromicrobiology, protein, protein nanowires

## Introduction

**Figure 1:**
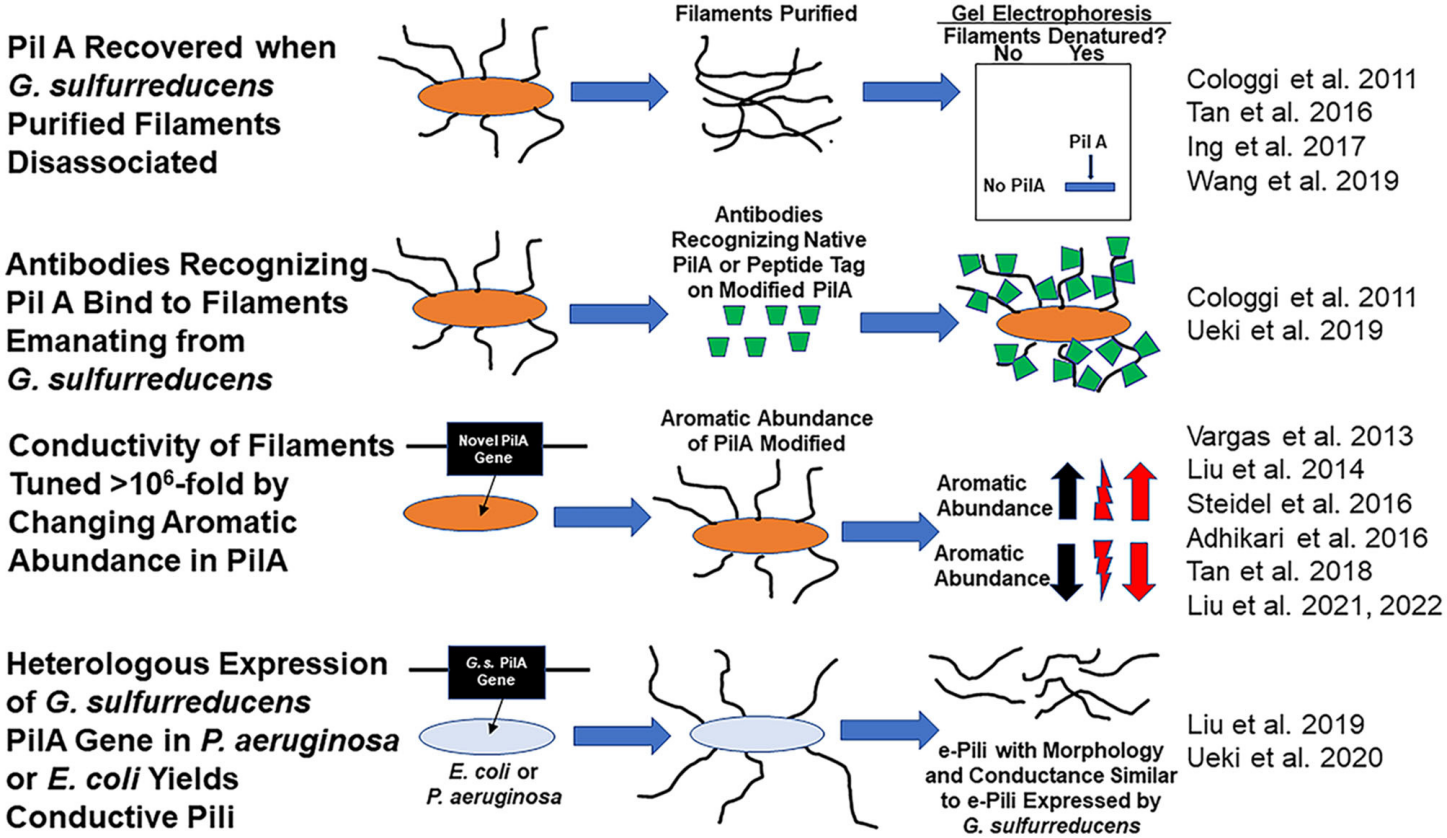
Evidence consistent with the hypothesis that *Geobacter sulfurreducens* expresses e-pili comprised of the pilin monomer, PilA and that PilA can be assembled into conductive filaments.

There is a debate whether *Geobacter sulfurreducens* produces electrically conductive pili (e-pili) from its pilin monomer, PilA, a protein encoded by gene GSU 1496. *G. sulfurreducens* assembly of the PilA into e-pili was proposed over a decade ago (Reguera et al., 2005). As detailed below, many subsequent studies have provided additional data consistent with this concept (Figure 1). However, Gu et al. have recently concluded that *G. sulfurreducens* does not express e-pili from PilA (Gu et al., 2021).

This is not a controversy over small details of the physiology of one microbe. *Geobacter* species play an important role in natural environments and biotechnologies. For example, *Geobacter* species are typically abundant in soils and sediments in which Fe(III) oxide reduction has a significant impact on the biogeochemical cycling of carbon, nutrients, and trace metals as well as in bioremediation (Lovley et al., 2011; Reguera and Kashefi, 2019; Lovley and Holmes, 2022). *Geobacter* species are also often abundant in soils and anaerobic digesters in which direct interspecies electron transfer (DIET) appears to be an important mechanism for methane production (Zhao et al., 2020; Lovley and Holmes, 2022). *Geobacter* and closely related species are often enriched on the anodes of electrodes harvesting electricity from organic matter and *G. sulfurreducens* generates the highest current densities of all known electroactive isolates (Lovley et al., 2011; Logan et al., 2019). Although other microbes, most notably *Shewanella* species, have been helpful for developing an understanding of key extracellular electron transfer mechanisms (Shi et al., 2016; Lovley and Holmes, 2022), there are no pure cultures that are as effective in Fe(III) oxide reduction, DIET, and current production as *G. sulfurreducens* and its close relative *G. metallireducens*.

Furthermore, if it were true that PilA cannot be assembled into conductive filaments, this would mean that attempts to develop new protein-based electronic materials based on concepts for electron transport along e-pili (Creasey et al., 2018; Dorval Courchesne et al., 2018; Gutermann and Gazit, 2018; Cosert et al., 2019; Roy et al., 2020) may be misguided. The reported heterologous expression of e-pili from PilA in *Pseudomonas aeruginosa* (Liu et al., 2019) or *Escherichia coli* (Ueki et al., 2020) for mass production of e-pili for the fabrication of electronics would require new, non-obvious explanations to describe how introducing *G. sulfurreducens* PilA confers the capacity for conductive filament expression. Other apparent accomplishments for electronics applications, also achieved simply by modifying the structure of PilA, such as tuning of the conductivity of *G. sulfurreducens* filaments or the introduction of novel binding sites on filaments to enhance sensor selectivity (Lovley and Yao, 2021), would also need reevaluation. The function of electronic devices for electricity generation (Liu et al., 2020b), sensing (Liu et al., 2020a; Smith et al., 2020), and neuromorphic memory (Fu et al., 2020, 2021) would need to be reconsidered.

## The Claim That Wild-Type *G. sulfurreducens* Does Not Express Filaments Comprised of PilA

Gu et al. (2021) conclude that *G. sulfurreducens* does not assemble PilA into pili because “Purified filament preparations from wild-type cells grown under these nanowire-producing conditions did not show either PilA-N or PilA-C using immunoblotting” (in Gu et al. the term PilA-N refers to the PilA protein encoded by gene GSU 1496). Yet just 2 years earlier the same lab reported that “we confirmed the presence of both PilA and OmcS with expected molecular weights of _~_6.5 kDa and _~_45 kDa, respectively, in our filament preparations using poly-acrylamide gel electrophoresis (SDS-PAGE), peptide mass spectrometry, and western immunoblotting” (Wang et al., 2019). Furthermore, the senior author of Gu et al. had also reported the recovery of PilA and OmcS from *G. sulfurreducens* filament preparations in another publication (Tan et al., 2016). It is important to recognize that these prior findings from some of the same investigators directly refute the Gu et al. hypothesis that wild-type *G. sulfurreducens* does not express filaments comprised of PilA. As detailed in the next section, there is also additional abundant evidence that wild-type *G. sulfurreducens* displays conductive filaments comprised of PilA.

Gu et al. did recover PilA-containing filaments from a mutant strain in which the gene for the outer-surface cytochrome OmcS was deleted (Gu et al., 2021). However, these filaments also contained another protein, and the filaments were poorly conductive. Gu et al. acknowledged that these hybrid filaments, which had a diameter of 6.5. nm, were an artifact produced only in the mutant strain; they were not expressed in wild-type *G. sulfurreducens*. As detailed below, no other study of *G. sulfurreducens* has observed 6.5 nm filaments emanating from *G. sulfurreducens* or in purified filament preparations. Such filaments were not even observed in other *omcS*-deletion mutants of *G. sulfurreducens* (Leang et al., 2010; Liu et al., 2022). Thus, the 6.5 nm PilA-containing filaments that Gu et al. report are an artifact, not replicated in other studies, and clearly have no relevance to the filament expression of wild-type *G. sulfurreducens*.

## The Evidence for e-pili Comprised of PilA

Many studies have provided substantial evidence that wild-type *G. sulfurreducens* expresses filaments comprised of PilA (Figure 1). For example, the Reguera lab eloquently demonstrated that: (1) the PilA pilin monomer was the only protein recovered from purified *G. sulfurreducens* filaments sheared from cells and (2) that intact filaments harvested from the cells reacted with a PilA-specific antibody (Cologgi et al., 2011). Several other laboratories subsequently demonstrated that PilA was a major protein in filaments recovered from *G. sulfurreducens* (Tan et al., 2016; Ing et al., 2017).

*G. sulfurreducens* PilA is assembled into conductive filaments, not only in *G. sulfurreducens*, but also in other microbes. Expression of the *G. sulfurreducens* PilA pilin monomer gene in *P. aeruginosa* (Liu et al., 2019) or *E. coli* (Ueki et al., 2020) yielded filaments with the same morphology and conductance as *G. sulfurreducens* e-pili.

Another observation that only seems explicable if e-pili are comprised of PilA is the dynamic tuning of pili conductivity by more than one million-fold that is possible simply by modifying the abundance of aromatic amino acids in the pilin monomer protein. For example, replacing the *G. sulfurreducens* PilA gene with the *G. metallireducens* PilA gene yielded filaments with the same 3 nm diameter of the wild-type *G. sulfurreducens* pili, but with a conductivity that was 5,000-fold higher than wild-type (Tan et al., 2017). The higher conductivity was attributed to a higher abundance of aromatic amino acids in the *G. metallireducens* pilin. Conversely decreasing the abundance of aromatic amino acids in the pilin, still yielded 3 nm diameter filaments, but with a conductivity 1,000-fold lower than wild-type (Adhikari et al., 2016).

Not only is there substantial evidence that *G. sulfurreducens* expresses conductive filaments comprised of the PilA pilin monomer, but also direct examination of filaments emanating from cells revealed that e-pili are the primary filaments that *G. sulfurreducens* produces. In one approach, synthetic pilin monomer genes that yield pilin monomers with peptide tags were expressed in *G. sulfurreducens* (Ueki et al., 2019). All the pili that these strains of *G. sulfurreducens* displayed reacted with antibodies that specifically bind to the peptide tags that were incorporated in PilA. The stoichiometry of antibody binding to pili could be tuned by controlling the relative quantity of synthetic pilin with tags vs. wild-type pilin expressed in strains containing genes for both pilin types (Ueki et al., 2019).

In an alternative approach, atomic force microscopy revealed that 90% of the filaments that *G. sulfurreducens* displayed had the same 3 nm diameter, morphology, and conductance as the conductive filaments produced when *E. coli* heterologously expressed *G. sulfurreducens* PilA (Liu et al., 2021). The other 10% of the filaments had a morphology and diameter consistent with filaments comprised of the *c*-type cytochrome OmcS. Replacing the PilA gene in *G. sulfurreducens* with a gene for a pilin monomer with reduced aromatic amino acid content yielded a strain in which over 90 % of the filaments emanating from the cells were 3 nm diameter pili, morphologically similar to the pili of the strain expressing PilA, but with 1,000-fold less conductance. The abundance and conductance of the filaments comprised of OmcS was unchanged. A similar predominance of 3 nm diameter conductive pili and then decreased pili conductance when PilA was replaced with a gene for an aromatic-poor pilin was observed in studies conducted in a strain of *G. sulfurreducens* in which the gene for OmcS was deleted (Liu et al., 2022). The finding that changing the aromatic abundance of the pilin protein specifically and dramatically changed the conductance of the 3 nm diameter filaments indicated that these filaments were comprised of pilin (Liu et al., 2021, 2022). Thus, multiple lines of evidence suggest that *G. sulfurreducens* displays conductive pili comprised of PilA and that these are the most abundant filaments emanating from cells.

## Importance of e-pili in Extracellular Electron Transfer

*G. sulfurreducens* requires its abundant e-pili for effective long-range extracellular electron transfer. The phenotypes of *Geobacter* strains that express poorly conductive pili provide the most direct evidence. Simply deleting the gene for PilA to prevent e-pili expression is not an appropriate approach because outer-surface *c*-type cytochromes that are also important for extracellular electron transfer are not properly localized to the outer surface in *pilA*-deletion mutants (Izallalen et al., 2008; Steidl et al., 2016; Liu et al., 2018). However, as noted above, *G. sulfurreducens* strains that express poorly conductive pili can be constructed by replacing the PilA gene with genes for pilins with a lower abundance of aromatic amino acids. These strains, which include *G. sulfurreducens* strains Aro-5, *G. sulfurreducens* strain Tyr3, *G. sulfurreducens* strain PA, and *G. metallireducens* strain Aro-5 express poorly conductive pili, while properly positioning outer-surface cytochromes on the outer cell surface (Vargas et al., 2013; Liu et al., 2014, 2021; Adhikari et al., 2016; Steidl et al., 2016; Ueki et al., 2018). None of these strains effectively reduce Fe(III) oxides or produce high current densities. *G. metallireducens* strain Aro-5 is an ineffective electron-donating partner for DIET (Ueki et al., 2018; Holmes et al., 2021).

The simplest explanation for these results is that the intrinsic conductivity of the wild-type e-pili is essential for effective extracellular electron transfer to Fe(III) oxides, other microbes, and through thick current-producing biofilms. *G. sulfurreducens* extracellular electron exchange is likely to rely on complex interactions between a suite of outer-surface *c*-type cytochromes, e-pili, and possibly other components (Lovley and Holmes, 2022). The phenotypes of strains expressing poorly conductive pili and cytochrome-deficient mutant strain phenotypes, as well as observations of cytochrome localization, demonstrate that cytochrome-based filaments alone cannot be the primary route for *G. sulfurreducens* long-range electron transfer (Lovley and Holmes, 2020, 2022).

## Conclusions

In conclusion, many studies have provided evidence that *G. sulfurreducens* expresses e-pili comprised of the pilin monomer PilA. It remains a mystery as to why Gu et al. (2021) did not recover filaments comprised of PilA from their strain of ‘wild-type' *G. sulfurreducens* when so many other studies, including several by the senior author of Gu et al., had previously found PilA in filament preparations. Furthermore, e-pili comprised of PilA can clearly be seen emanating from cells of *G. sulfurreducens*. Other microbes can heterologously express the *G. sulfurreducens* PilA and assemble it into the same type of e-pili found in *G. sulfurreducens*. Consistent with these observations, *G. sulfurreducens* nanowire conductivity is readily tuned simply by changing the abundance of aromatic amino acids in the pilin expressed. Expression of poorly conductive pili has demonstrated the importance of e-pili in Fe(III) oxide reduction, electron transfer to other microbial species, and for generating high current densities in bioelectrochemical systems. Therefore, at present the preponderance of evidence is that e-pili, comprised of PilA, not only exist, but are an important feature in *Geobacter* extracellular electron exchange. The pilins and archaellins of phylogenetically distinct bacteria and archaea are assembled into conductive filaments and it seems likely that e-pili and e-archaella are spread throughout the microbial world (Walker et al., 2018, 2019, 2020; Bray et al., 2020; Lovley and Holmes, 2020).

## Author Contributions

The author confirms being the sole contributor of this work and has approved it for publication.

## Conflict of Interest

The author declares that the research was conducted in the absence of any commercial or financial relationships that could be construed as a potential conflict of interest.

## Publisher's Note

All claims expressed in this article are solely those of the authors and do not necessarily represent those of their affiliated organizations, or those of the publisher, the editors and the reviewers. Any product that may be evaluated in this article, or claim that may be made by its manufacturer, is not guaranteed or endorsed by the publisher.
